# Upconversion nanoparticles and its based photodynamic therapy for antibacterial applications: A state-of-the-art review

**DOI:** 10.3389/fchem.2022.996264

**Published:** 2022-10-04

**Authors:** Hanlin Lv, Jie Liu, Ying Wang, Xiaomin Xia, Ying Li, Wenxue Hou, Feng Li, Lantian Guo, Xue Li

**Affiliations:** ^1^ Department of Stomatology, The Affiliated Hospital of Qingdao University, Qingdao University, Qingdao, China; ^2^ School of Stomatology, Qingdao University, Qingdao, China; ^3^ College of Automation and Electronic Engineering, Qingdao University of Science and Technology, Qingdao, China

**Keywords:** conjugated nanoparticle, upconversion, photodynamic therapy, antibacterial, near-infrared

## Abstract

Major medical advances in antibiotics for infectious diseases have dramatically improved the quality of life and greatly increased life expectancy. Nevertheless, the widespread and inappropriate exploitation of antibacterial agents has resulted in the emergence of multi-drug-resistant bacteria (MDR). Consequently, the study of new drugs for the treatment of diseases associated with multi-drug-resistant bacteria and the development of new treatments are urgently needed. Inspiringly, due to the advantages of a wide antimicrobial spectrum, fast sterilization, low resistance, and little damage to host tissues and normal flora, antibacterial photodynamic therapy (APDT), which is based on the interaction between light and a nontoxic photosensitizer (PS) concentrated at the lesion site to generate reactive oxygen species (ROS), has become one of the most promising antibacterial strategies. Recently, a burgeoning APDT based on a variety of upconversion nanoparticles (UCNPs) such as PS and near-infrared (NIR) light has been fully integrated in antibacterial applications and achieved excellent performances. Meanwhile, conjugated nanoparticles have been frequently reported in UCNP design, including surface-modified PS conjugates, antibiotic-PS conjugates, and dual or multiple antibacterial modal PS conjugates. This article provides an overview of the state-of-the-art design and bactericidal effects of UCNPs and their based APDTs. The first part discusses the design and mechanisms for UCNPs currently implemented in biomedicine. The second part focuses on the applications and antimicrobial effects of diverse APDT based on UCNPs in antibacterial-related infectious diseases.

## 1 Introduction

At the beginning of the 20th century, infectious diseases caused by pathogenic bacteria were among the leading causes of death and presented growing challenges to health security and human progress (Cohen, 2000; Nii-Trebi, 2017). The emergence of antimicrobial agent was primarily responsible for the improvement in cure rate and decreases in mortality for infection diseases (Huh and Kwon, 2011). However, with the increasing abuse of antibiotics in treatment and more and more infections caused by multi-drug-resistant bacteria (MDR), resistance to antibiotics has reached a rather tedious situation (Metlay et al., 2006; Martinez and Silley, 2010; Fair and Tor, 2014). Even now, first-line antibiotics currently used are invalidated in clinical therapy (Blair et al., 2015). Therefore, discovering new drugs or inventing an efficient and nontoxic bactericidal treatment has been constantly attempted to match up to the pathogenic bacteria’s speediness and frequency variation.

Unprecedented superiority of nanomaterials and novel mechanisms of action have been introduced to establish a potent platform that is capable of eliminating pathogenic bacteria efficiently without the risk of drug resistance (Cieplik et al., 2018a; Zhang et al., 2018). Antibacterial photodynamic therapy (APDT), a promising alternative approach, has been believed to meet the pressing need. Light with an appropriate wavelength and a biosafety photosensitizer (PS) with a matching absorption spectrum make up the APDT (Karner et al., 2020). After the activation, usually by ultraviolet (UV) or visible range emission light, the excited PS could undergo a chemical reaction with oxygen that generates reactive oxygen species (ROS), including singlet oxygen (^1^O_2_) and a hydroxyl radical around rapidly (Karimi et al., 2017; Xu et al., 2017). The above oxidative burst could kill bacteria selectively and efficiently (Cieplik et al., 2015; Cieplik et al., 2018b). This therapy method has been investigated in abundant *in vitro* and *in vivo* studies and often reached an inactivation ratio of more than 5 log10 of CFU (colony forming units) (Boyce and Pittet, 2002). In particular, a lot of evidence suggests that APDT has emerged as an effective modality for MDR infections such as *S. aureus*, *A. baumannii*, *Klebsiella pneumoniae*, *E. coli*, and so on (Zhang et al., 2018; Jia et al., 2019; Li et al., 2020a; Tosato et al., 2020). However, the limited penetration depth in biological tissues ranged from 60 µm to several millimeters of UV or visible light and the toxicity of UV in particular weakened APDT therapeutic efficacy for deep-tissue infection to a great degree (Sharma et al., 2011; Hou et al., 2016; Liang et al., 2016).

Judging from the limitations highlighted above, among multiple nanomaterials, upconversion nanoparticles (UCNPs) that are usually doped with lanthanide rare earth elements have attracted wide attention in biomedical diagnosis and treatment (Idris et al., 2015a). The core principle of UCNPs is based on the anti-Stokes shift luminescence mechanism, which could covert near-infrared (NIR) light into UV or visible light in keeping with the activation wavelength of existing PS. Compared with UV and visible emission light, NIR light, whose wavelengths range from 650 to 1,350 nm, has better penetration depth in the soft tissue (Weissleder, 2001). Along with the superb penetration depth of NIR excitation, UCNPs have represented an enormous preponderance of PS loading nanoplatforms in NIR-triggered APDT to overcome the drawbacks mentioned above. As a result, traditional and clinically effective PS could continue to be used for deep-tissue therapy. Moreover, UCNPs have high chemical stability and could be easily functionalized by linking specific peptides, antibiotics, small-molecule drugs, or metallic elements to extend biological applications with high sensitivity and selectivity (Rao et al., 2017). Conjugates are a commonly used method for improving nanomaterial performance in antitumor ability, biosafety, drug delivery, and other properties in biomedical applications (Tan and He, 2021; Wang et al., 2021). Conjugated UCNPs with polymeric composite, antibiotics, silica coating, PS, and other functionalized nanoparticles were also constructed to increase antibacterial activity or achieve multiple strategy sterilization.

Clearly, in the future, for the UCNPs designed for antibacterial or antitumor application, conjugation strategy will be continually used to gain more effective function or synergistic effect. In recent years, plenty of significant reviews on UCNPs and their based APDT treatment have been announced, mainly focusing on the catalysis, luminous performance, bioimaging applications (e.g., MRI, X-ray CT imaging, photoacoustic (PA) imaging, NIR thermal imaging, and upconversion luminescence imaging), and tumor treatment (e.g., chemotherapy and radiotherapy) (Cheng et al., 2013; Wang et al., 2013; Kim et al., 2017; Wang et al., 2017; Duan et al., 2018; Dong et al., 2019). Nevertheless, the systematic antibacterial and anti-infection effects of UCNPs and its based APDT treatment with different material designs were rarely reviewed. Therefore, this review began with a brief overview of the current state of antibacterial and anti-infective therapy, followed by a preliminary outline of the antibacterial mechanism of UCNPs and the APDT treatment based on it. In addition, this article presented a comprehensive overview of novel achievements in nanomaterial compositions and the antibacterial applications of their based APDT against infectious diseases. We hope that this article will provide guidance for developing a new anti-infection strategy in the future.

## 2 Information sources and search strategy

The search was conducted through the PubMed, Web of Science, and Google Scholar databases using standardized methodological filters up to September 2015 across all databases with no time restrictions. Only English version articles were selected. Search strategies were mainly constructed based on these keywords: “upconversion”, “photodynamic therapy”, and “antibacterial” as follows: (“upconversion” OR “upconversion nanoparticles”) AND (“photodynamic therapy”) AND (“antibacterial” OR “bacteria” OR “infection”). In addition, the retrieved papers were manually selected to find relevant articles.

## 3 Antibacterial mechanism of antibacterial photodynamic therapy

A typical APDT process is made up of three inseparable parts: PS (light-absorbing molecule), a light source with a specific spectrum, and dissolved oxygen in cells (Hu et al., 2018). After administration of PS and external light irradiation, PS-absorbed photons then translate from the ground state to a temporary singlet state. After that, via “intersystem crossing” or spin–flip of the HOMO electron singlet state, PS could produce relatively steady triplet state species, which could generate a large amount of ROS in a short time (Hamblin, 2018). ROS could cause bacteria to be damaged in a variety of ways and then control infection (Vaishampayan and Grohmann, 2021). Benefiting from its unique antibacterial mechanism, APDT does not lead to drug resistance in bacteria, which gives it a huge advantage over conventional antibacterial drugs (Bekmukhametova et al., 2020).

**FIGURE 1:**
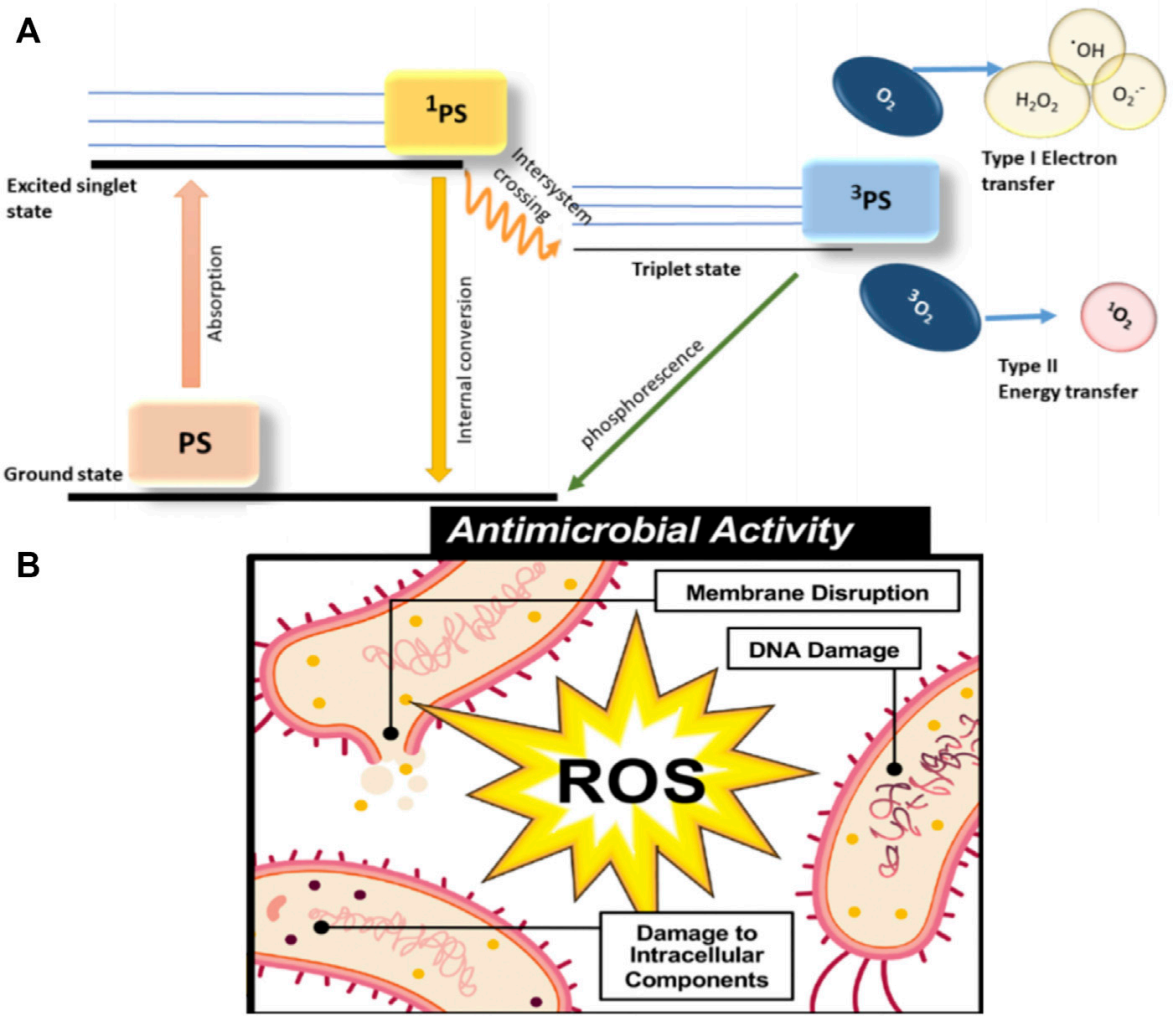
**(A)** Illustration of the photochemical mechanisms of PDT types I and II. ^1^PS, PS in a first excited state; ^3^PS, triplet-state PS;·OH, hydroxyl radical; O^2−^, superoxide anion; and H_2_O_2_, hydrogen peroxide. PS reaches the ^1^PS by absorbing energy, and a part of ^1^PS is converted to ^3^PS by intersystem crossing, and the other part is converted back to the ground state by internal conversion. Triplet PS produces ROS in two ways: Type Ⅰ electron transfer; Type II energy transfer. After that, ^3^PS goes back to the ground state (PS) through phosphorescence (Ghorbani et al., 2018). **(B)** Illustration of the three ROS antimicrobial mechanisms: Membrane disruption; DNA damage; and damage to intracellular components (Knoblauch and Geddes, 2020).

The pathways of ROS generation could be divided into two types (Figure 1A). Type Ⅰ showed that the excited PS could interact with the cellular membrane directly and then lead to electron or hydrogen shifting between PS and substrate, which could release hydroxyl radicals (·OH), superoxide anion (O_2_^−^), and hydrogen peroxide (H_2_O_2_). Type Ⅱ demonstrated that the energy from irradiation could be transferred from molecular oxygen to highly oxidative properties. According to past research, Type II in the formation of hydroxide was extremely crucial to the therapeutic effect of APDT. Interestingly, Type Ⅰ usually prevails in the dominant position in low-oxygen environments, and both pathways could occur simultaneously (Kwiatkowski et al., 2018). ROS can harm bacteria in a variety of ways (Figure 1B), including DNA destruction, lipid peroxidation, enzymatic system inhibition, and protein denaturation. ROS could not only induce the destruction of normal functions of DNA in microbial cells, but also result in the peroxidation of lipid in the cell membrane and the disruption of the normal function of the lipid layer. The abovementioned changes could block the working of membrane-located receptors and proteins that lead to cell perforation, losing cytosolic contents, and a decrease in enzyme activity (Memar et al., 2019). However, there are still some limitations in APDT, such as the limited tissue penetration and underlying risk of cytotoxicity due to the common use of UV or visible light as an exciting light (Ovais et al., 2020).

This section may be divided by subheadings. It should provide a concise and precise description of the experimental results, their interpretation, and the experimental conclusions that can be drawn.

## 4 Upconversion luminescence mechanisms

**FIGURE 2:**
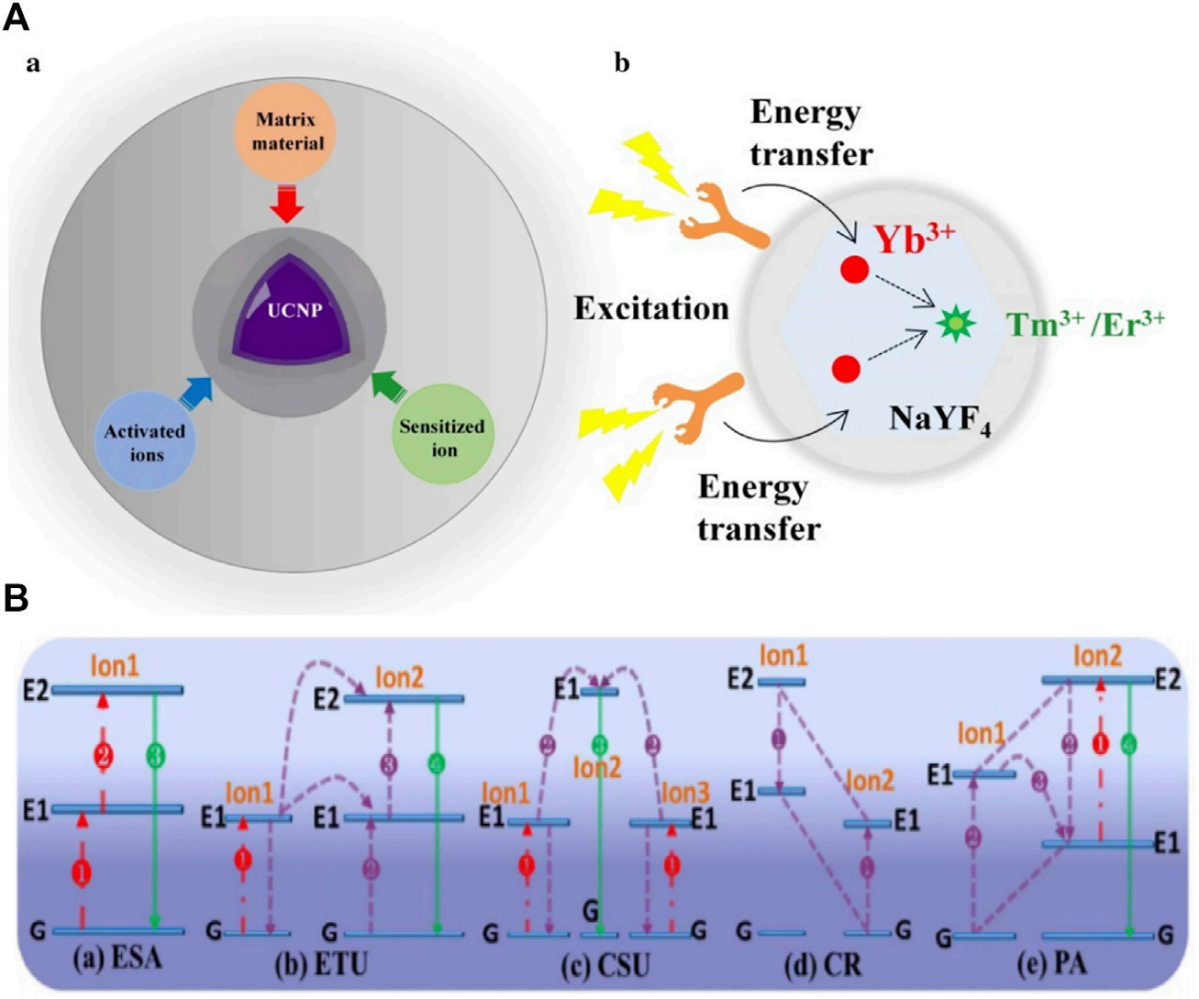
**(A) (a)** The basic composition of UCNPs: matrix material, activated ions, and sensitized ion; **(b)** schematic illustration of the mechanism of organic dye-sensitized UCNPs (Liang et al., 2020a). **(B)** Excited-state absorption (ESA), energy transfer upconversion (ETU), cooperative sensitization upconversion (CSU), cross relaxation (CR), and photon avalanche (PA). The red, violet, and green lines, respectively, represent photon excitation, energy transfer, and emission processes (Chen et al., 2014).

Recently, UCNPs have drawn extensive attention because of their higher stability, ideal signal-to-noise ratio, and ability to be easily activated by low-energy photons (Sun et al., 2019). For instance, the role of UCNPS in improving photoelectric conversion efficiency has received widespread attention in the area of solar cells (Guo et al., 2021). UCNPs are distinct optical nanomaterials and the most representative is lanthanide-doped UCNPs, which exhibit favorable photo-bleaching resistance, deep-tissue penetration performance, and minimal photo-damage (Liang et al., 2020b). The structure of UCNP comprises an inorganic photostable host matrix, a sensitizer, and an activator (Figure 2A). The activated ions provide luminescent centers, the sensitized ions absorb NIR light, and the matrix provides a crystalline host lattice structure. UCNPs are usually prepared from rare earth fluorides doped with transition metal (3, 4, 5 days), lanthanide (4f), or actinide (5f) ions. Trivalent lanthanide ions are the most ideal doping materials at present (Yao et al., 2020). Not all UCNPs are suitable for the applications in APDT. Many factors, such as production cost, material biotoxicity, UCL efficiency, and so on, should be considered in a variety of UCNPs. UCNP emission wavelength can be modulated precisely by choosing the type of lanthanide dopants in a UCNP in the right way (Zhang and Zhang, 2021). Therefore, by adjusting the type of doping material, UCL could emit ultraviolet and visible light that excites the traditional APDT system. NaYF_4_ is considered one of the most effective matrix materials in biological implications due to its advantages of high chemical stability and low photon energy. Yb^3+^ or Nd^3+^ is the most commonly used sensitizer ions, has larger absorption cross-sections, and can completely transfer the absorbed energy to adjacent excited ions in the crystal lattice. The most common activating ions is the lanthanide ion Yb^3+^. Activated ions are essential to the entire UC emission process. The activator is responsible for emitting visible light and UV light from NIR light absorbed by sensitized ion conversion, making Er^3+^ or Tm^3+^ the best candidates because of their long-lived intermediate energy states and because it is convenient to move them from the excitation of their intermediate state to the higher state (Yan et al., 2017).

There are five types of upconversion luminescence (UCL) mechanisms (Figure 2B) of upconversion nanoparticles: 1) excited-state absorption (ESA), 2) energy looping/photon avalanche (PA), 3) energy transfer upconversion (ETU), 4) cooperative sensitization (CS), and 5) energy migration upconversion (EMU). Notably, PA is rarely found in lanthanide materials (Lin et al., 2021).

ESA is the continuous absorption of one or more photons from the ground state to the intermediate excited state, thus obtaining UC emission (Zhou et al., 2015). ETU is similar to ESA and could play a key role in upconversion luminescence due to its high UCL efficiency (Mahata et al., 2017). The sensitizer ion (ion 1) with a larger absorption cross could reach the excited level state by absorbing a pumping photon. Then, energy could be later transferred to the neighboring activator ion (ion 2) in the ground state or intermediate state. The upconversion emission is then released once the excited activator returns to the lower energy or ground state. The ETU generally requires that the sensitizer and activator ions be at an appropriate distance to realize efficient non-radiative energy transfer (Li et al., 2020b).

EMU and CS require multiple centers in the sensitization or luminescence process, which involve cooperative effects. In the case of cooperative sensitization, two excited ions (ions 1 and 2) absorb one photon to generate their excited levels and then together, transfer the energy to another ion (ion 3) to upgrade its excited-state level. In the case of cooperative luminescence, two excited interacting ions (ions 1 and 2) could absorb one photon and cooperate in producing the emission (Li et al., 2020c; Kandoth et al., 2021; Li et al., 2021).

**FIGURE 3:**
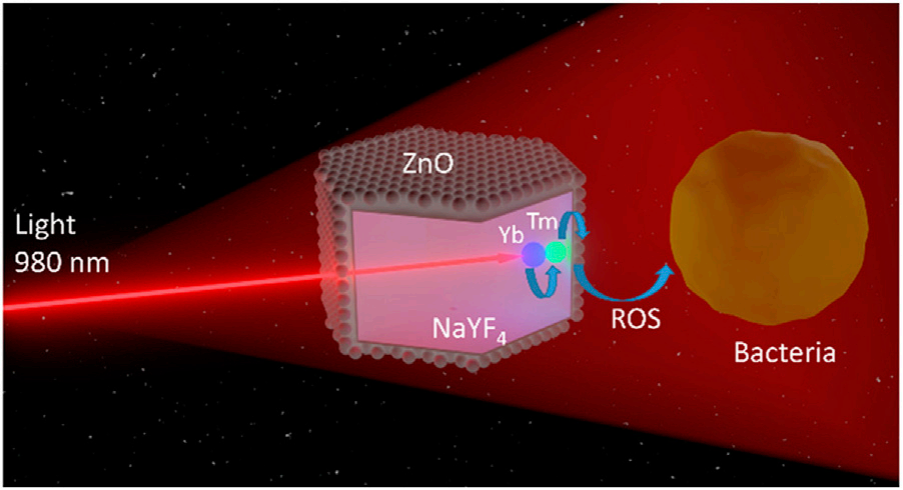
The dopant Yb^3+^ and Tm^3+^ ions convert 980 nm of NIR light into UV light. The ZnO in the outer layer of the core-shell structure absorbs UV light to produce ROS and achieve the antibacterial effect (Karami et al., 2021).

The transition of UV or visible light from long wavelength excitation radiation is a unilinear anti-Stokes luminescence process. UCNPs absorb two or more low-energy photons and then emit higher energy light, which is different from the common Stokes luminescence type. By virtue of the long-lived and real ladder-like intermediate levels of rare-Earth ions, UCNPs could effectively convert NIR light into a higher energy emission photon, thus NIR to UV and visible light, which enables PS to be excited by deep-tissue bioimaging light (Sivasubramanian et al., 2019). Hence, using UCNPs combined with NIR, the undesirable aspects of UV- and visible light-activated material can be accordingly addressed. As an example, Figure 3 illustrates the APDT mechanism of NaYF_4_: Yb^3+^/Er^3+^@ZnO (Karami et al., 2021).

## 5 Upconversion nanoparticles in antibacterial photodynamic therapy

This part mainly reviewed several different designs of UCNPs in recent years for APDT. As mentioned before, to convert UV-excited PS into NIR or visible light, UCNP conjugates are a feasible measure. Furthermore, hydrophobic ligands on the surface of UCNPs impede their dispersion in an aqueous environment and in biological applications due to the common fabrication routes (Idris et al., 2015b). Therein, surface modification is common in UCNPs to improve the hydrophilicity, biocompatibility, and PS-carrying capacity. There are five normal methods of surface modification: ligand removal, ligand exchange, ligand attraction, ligand oxidation, and surface silanization (Sedlmeier and Gorris, 2015). Among the many methods, surface silica coating and polymeric coating are the two most commonly used in APDT. These PS and surface-modified UCNPs conjugates cannot only generate non-ultraviolet excitation, but also have better biocompatibility and hydrophilicity.

### 5.1 Surface silica coating

Silica (SiO_2_) is widely used in the surface modification of UCNPs due to its great biocompatibility, optical transparency, tunable pore size, and chemical stability. In addition, the silica layer can carry other functional groups more easily, such as −SH, −NH_2_, and −COOH (Li et al., 2022).

Tou et al. (2017) have synthesized a core–shell–shell structure UCNPs by sequentially coating SiO_2_ and ZnO nanoparticles on the surface of NaYF_4_: Yb^3+^/Tm^3+^, enhancing the water-solubility and structural uniformity of the nanoparticle (UCNPs@ZnO). Under the 980-nm NIR condition, ZnO was the PS to generate ROS. In the antibacterial experiments, the MIC values of the UCNPs in *E. coli*, *S. aureus*, and *C. albicans* experiments were far below the standard value.

Tan et al. (2018) designed similar double-layer core-shell UCNPs, aiming to improve the visible light absorption of ZnO. The co-doped material Zn_1-x_Mn_xO_ as the outer layer could increase the efficiency of the photocatalytic activity of ZnO, thereby improving the absorption capacity and exhibiting a higher inhibitory effect on *E. coli* and *S. aureus* cells, which has been proved by MIC experiments.

A core-shell UCNP coated with mesoporous silica has been reported by Grüner and coworkers. They loaded silicon (IV)2,9,16,23-tetra-tert-butyl-29H, 31H-phthalocyanine dihydroxide (SiPc) onto the surface of a mesoporous silica shell. *E. coli* was eradicated, and *S. aureus* was significantly reduced, whereas under 978 nm irradiation, the inhibition toward Gram-positive *S. aureus* was not apparent. It is possible that the thickness of the mesoporous silica layer and the peptidoglycan layer of Gram-positive bacteria contribute to this phenomenon. Further studies should be carried out on that mesoporous silica coating to enhance ROS diffusion (Grüner et al., 2018). Zhang et al. developed silane-coated UCNPs (NaYF_4_: Yb^3+^/Tm^3+^/Mn UCNPs). Chlorine 6(Ce 6) was loaded onto the hydrophobic layer. Mn doping could enhance red region emission and then improve PDT efficiency. Several different concentrations of Mn-doped UCNPs were established to identify the Mn-doping enhancement to APDT. Three main periodontal pathogens have been chosen (*S. sanguinis*, *P. gingivalis*, and *F. nucleatum*). Live/dead stain images clearly illustrated that with the increase of Mn doping, the antibacterial effect becomes more effective. CFU experiments further confirmed this enhancement, under 980 nm NIR irradiation, 10% Mn doping groups and Mn free doping UCNPs (NaYF_4_@Ce6@silane) showed similar inhibition (within 1 log). With the increase of Mn doping, 30% Mn-doping groups showed more than 2log CFU reduction which demonstrated good sterilization activity. The inhibition of extracellular polymeric substances (EPSs), which is extremely important to pathogen resistance, has been considered. The EPS reduction in several groups exhibits similar results. Periodontitis is a serious oral disease. Meanwhile, the complicated structure of the periodontal pocket and dental plague perplex the APDT in periodontitis treatment. In this work, molar tooth samples were used to culture pathogen biofilm and simulate human oral conditions. However, there is no *in vivo* experiment to further confirm the treatment effect of periodontitis in animal models (Zhang et al., 2019).

### 5.2 Polymeric coating

Li et al. (2016) reported a PAA surface coated with UCNPs@PFVCN and its inhibition to *E. coli.* PFVCN acted as a PS and increased the FRET (fluorescence resonance energy transfer) efficiency that may produce more ROS during laser treatment. The CFU reduction showed that under 980 nm NIR laser irradiation for 30 min, 90% of bacterial cells were killed. Fluorescence imaging tests further confirmed the sterilization activity.

Liu et al. first developed a NIR-triggered APDT for extensively drug-resistant *Acinetobacter* baumannii (XDR-AB), which is a vital bacterium for hospital infection. Free-ligand LiYF_4_: Yb^3+^Er^3+^ UCNPs are coated with PVP (polyvinyl pyrrolidone) to load RB (Rose Bengal) as the PS. In this report, *in vitro* and *in vivo* antimicrobial experiments of UCNPs-PVP-RB were organized to measure the APDT efficiency for XDR-AB. The *in vitro* test indicated that upon 980 nm NIR 10 min, the 50 μg ml^−1^ UCNPs-PVP-RB induced a 4.72 log10 CFU reduction, which was almost equal to the effect of extremely overdosed polymyxin B (4.90 log10). In the *in vivo* test, the recovery time and the HE-stained tissue slices of the XDR-AB infected wound on the mice’s back proved the excellent antibacterial effect of these UCNPs. Furthermore, they compared 980 and 550 nm light-triggered APDT *in vitro* on 5-mm thick pork tissue and showed that NIR light was superior for treating deep-tissue infections (Liu et al., 2020a).

Titanium dioxide nanoparticles have good biocompatibility and stability and could produce ROS under UV conditions. Qi and coworkers designed β-NaYF_4_: Yb^3+^/Tm^3+^ UCNPs@TiO_2_ with the coating of PVP. With the 980-nm NIR light, the viability of three main periodontal pathogens (e.g., *S. sanguinis*, *P. gingivalis*, and *F. nucleatum*) has been greatly reduced compared with the commercial APDT drug (Qi et al., 2019).

### 5.3 Antibiotics or antibacterial materials assembled upconversion nanoparticles in antibacterial photodynamic therapy

**FIGURE 4:**
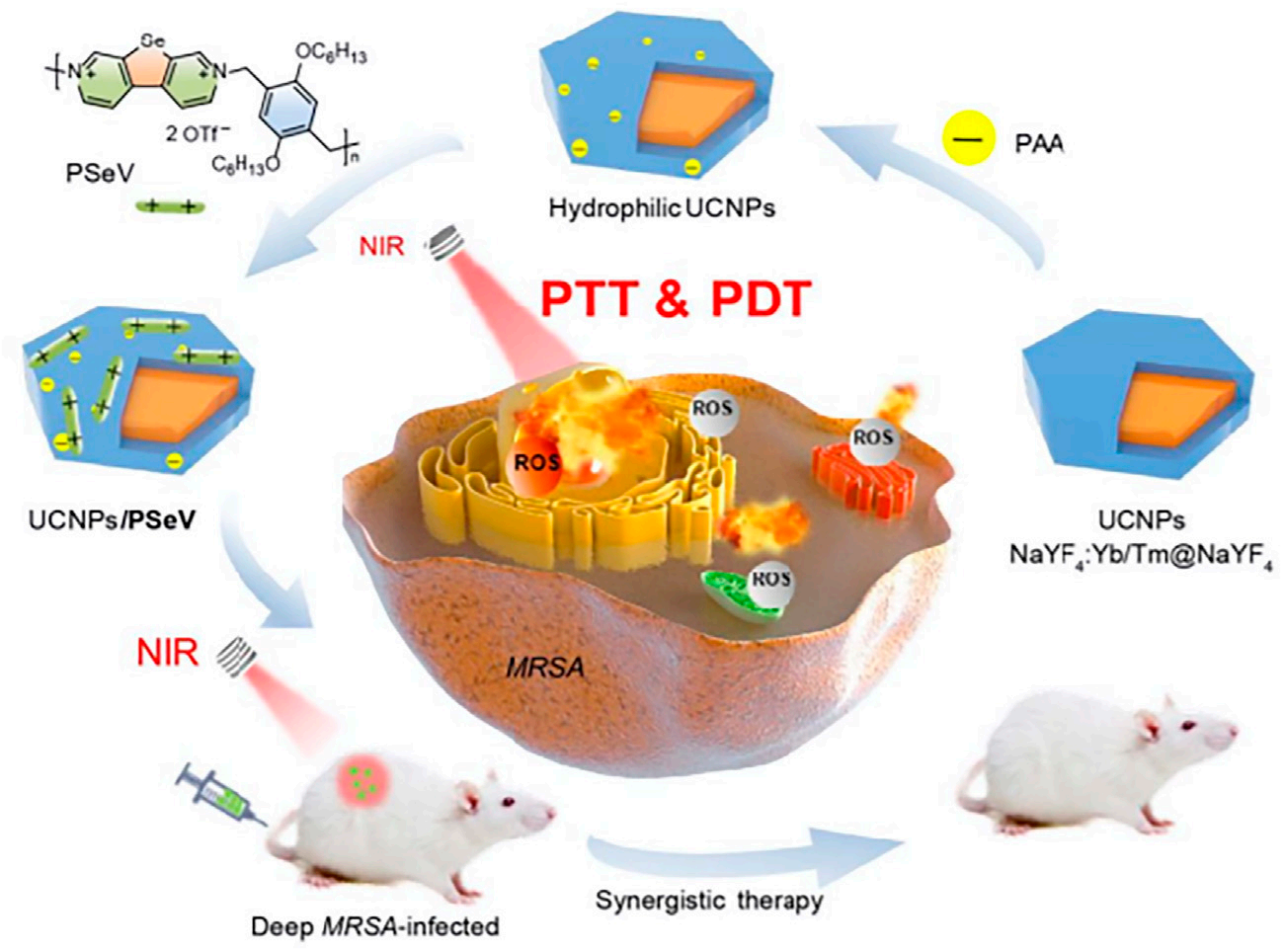
The PAA surface-modified UCNPs were assembled with PSeV to formulate a PTT and PDT synergistic conjugates. Photothermal and photodynamic effects combined to kill MRSA in deep tissue with NIR light (Zhou et al., 2020).

Although drug resistance and side effects limit the use of antibiotics in current anti-infection treatments, antibiotics are still one of the most effective drugs for treating bacterial infections. Some inorganic materials such as Ag^+^ and Cu^2+^ have excellent antibacterial activity, and they have also been maturely used in infection treatment. Some attempts to combine antibiotics or other antibacterial drugs with PS that developed UCNPs-PS-drug conjugates have achieved good results (Hou et al., 2020). After 10 min of NIR light irradiation, 8.33 × 10^6^ ml^−1^UCNPs@SiO_2_(MB)@AgNCs had a 100% bacteria killing rate for both *E. coli* and *S. aureus*. In dark conditions, this nanomaterial also exhibited an antibacterial effect to some extent, which means these silver-coated UCNPs have a dual-mode synergistic sterilization effect. The toxicity of heavy metal ions cannot be ignored. In this article, the biodiversity analysis was lacking. Thus, the ideal concentration of UCNPs@SiO_2_(MB)@AgNCs *in vitro* experiments could be limited due to the toxicity of silver ions or MB (Liu et al., 2020b). Considering the potential risk of noble ions, assembling a low-toxicity antibacterial composite seems to be an ideal choice. For instance, surface coating with high-biosecurity antibacterial material, such as chitosan, was also used to enhance the antibacterial efficiency of APDT and reduce its toxicity. Liu et al. created curcumin-assembled UCNPs that have a nearly 100% MRSA elimination ratio *in vitro*. Furthermore, a post-surgery MRSA infection model with SD rats has been established. In an *in vivo* test, 80% of MRSA cells have been eradicated in rats’ deep joint infection areas, which demonstrates high antibacterial activity in deep-tissue infection as shown in Figure 4 (Liu et al., 2018).

Li et al. (2017) reported an APDT system based on cationic N-octyl chitosan (OC)-coated UCNP loaded with the photosensitizer zinc phthalocyanine (OC-UCNP-ZnPc). Cationic compounds could interact with the bacteria’s negatively charged cell membrane and increase cell permeability or prevent necessary nutrient exchange in bacterial cell membranes. Furthermore, cationic compounds have high specificity for both Gram-negative and Gram-positive bacteria, which can increase the specificity of UCNPs to bacteria. *In vitro* and *in vivo* tests demonstrated powerful antibacterial properties against methicillin-sensitive *S. aerues* (MSSA), methicillin-sensitive *Staphycoccus epidermidis* (MSSE), *E. coli*, *P. aeruginosa*, and four drug-resistant strains of these bacteria at the same concentration (125 μg ml^−1^) and 980 nm laser irradiation for 10 min. The power density of the 980 nm laser is 0.4 W cm^−2^. The viability of MSSA was completely inhibited at 250 μg ml^−1^. Due to the difference in storm structure between Gram-positive bacteria and Gram-negative bacteria, Gram-positive bacteria were more sensitive to APDT. The MTT assay was used to determine the cytotoxicity of normal human liver cells L02 and normal breast cells HCL-100. The results indicated that even at 800 μg ml^−1^, cell activity is still more than 80%.

Multiple mode-based synergistic antibacterials were also reported, which combined the APDT mode with photothermal therapy (PTT) or sonodynamic therapy (SDT). These conjugates were loaded with PS and related functional particles. Synergistic therapy strategies have also been applied in antitumor field. Xu and coworkers developed a biodegradable copper/manganese silicate nanosphere (CMSN)-coated UCNP conjugate system for CDT (chemodynamic therapy)/PDT synergistic antitumor therapy with NIR light (Xu et al., 2020). To achieve single-wavelength light-triggered PDT-PTT, Zhou et al. built poly (selenoviologen) (PSeV)-assembled UCNPs (NaYF_4_: Yb^3+^/Tm^3+^@NaYF_4_). Under 980 nm NIR irradiation for 4 min, 2.5 × 10^−6^ M UCNPs/PSeV eradicated 98.3% of MRSA cells, whereas the individual bacterial killing ratio of UCNPs and PSeV was far below that of the synergistic effect (Zhou et al., 2020). Yin et al. (2014) designed (UCNPs/MB/CuS) combined PDT with PTT to obtain a high therapeutic effect via synergistic effects. Zhao et al. (2020) designed UCNPs@mSiO_2_ (RB)-AgNPs through PDT, SDT, and nano silver ions, three mechanisms to kill methicillin-resistant *Staphylococcus aureus*. Under 980 nm irradiation and supersonic for 10 min, *in vitro* tests suggested that the killing efficiency of 45 μg ml^−1^ UCNPs@mSiO_2_ (RB)-AgNPs has reached 100%. Both the PDT and SDT experiments showed that the two have synergistic effects. The released silver ions not only enhanced the instant antibacterial effect under PDT and SDT treatment but also showed long-term bacterial inhibition without PDT or SDT treatment. The material also showed strong antibacterial activity in extended incubation experiments, both in dark conditions and PDT + SDT treatments, which showed brilliant long-term antibacterial stability.

## 6 Limitations for upconversion nanoparticle-based antibacterial photodynamic therapy

Although UCNPs have been applied in many fields and shown huge potential, there are still many limitations that need to be improved and discussed in future research. The crystal structure, dopant ions, and surface functionalization of UCNPs with different capping agents strongly influence the UCL process for their practical use. In addition, nanoparticle size also has a great effect on the material. Smaller UCNPs have higher endocytosis efficiency but also possibly reduce UCL efficiency (Borse et al., 2022). Ultrasmall-sized (such as < 5 nm) UCNPs have poor UCL efficiency. However, large UCNPs cannot be eliminated by the kidney, and the elimination is mainly through the biliary tract, but this route needs more time and has a potential risk of hepatotoxicity (Chen et al., 2020).

Another noteworthy phenomenon is the overheating of biological tissues. Well-designed UCNP-based PTT systems could be used in the antitumor or antibacterial area. However, overheating due to the absorption of 980 nm NIR light by water may cause damage to normal soft tissues (I et al., 2020). 808 nm NIR light-triggered PDT systems have been reported to overcome this problem (Lee et al., 2020). But shorter excitation wavelengths mean less soft-tissue penetration (Chan et al., 2022). It has been reported that biological-window II (1,000–1700 nm) has a good application prospect in high-resolution imaging of deep tissue (Xiang et al., 2021). Furthermore, despite many reports of cytotoxicity of UCNPs within days, *in vitro* tests have shown promising results. Considering the complexity of many UCNP systems, the long-term stability and biosafety of these nanoparticles need to be seriously investigated. More research is also needed into the effects of UCNPs on the nervous and immune systems in humans (Jethva et al., 2022).

Species of bacteria also influence APDT efficiency. The special cell wall structure of Gram-negative bacteria makes it more difficult to be killed by ROS than Gram-positive bacteria. The membrane’s external face contains all the LPS, whereas the internal face contains most of the phospholipids. In contrast, Gram-positive bacteria have a cell wall that consists of a cytoplasmic membrane surrounded by a layer of relatively porous peptidoglycan and lipoteichoic acid that facilitates penetration of the photosensitizers into the inner membrane (de Siqueira et al., 2022). As mentioned before, especially in the single-mode APDT system, the elimination effect of UCNPs against Gram-negative bacteria such as *E. coli* was generally significantly lower than that against Gram-positive bacteria such as *S. aureus*. Thus, in the treatment of deep Gram-positive bacterial infections, the efficiency of APDT may be reduced. Besides, in addition to *in vitro* experiments, more attention should be paid to animal experiments, especially in deep-tissue infection models. Improving UCNP doping, light sources, and using multimodal synergistic antimicrobial strategy will be the main research directions in the future (Song et al., 2021).

## 7 Conclusion

UCNPs have attracted great attention in recent years because of their UCL properties. A number of previous studies have reported its antitumor, microbiological, and bioimaging properties. In this article, APDT on the basis of UCNPs has been briefly reviewed by the components, mechanisms, utilization, and current situation. It appears that antibacterial photodynamic therapy will be more closely integrated with other antimicrobial therapies in the future to achieve higher bactericidal efficiency and lower damage to the human body. NIR light-triggered APDT damage to normal tissue is negligible, and its deep penetration guarantees a stronger therapeutic effect than ultraviolet light. Certainly, toxicity to the mammalian nervous system and other tissues should be further studied, and light conversion efficiency and tissue penetration depth should also be further improved. To date, UCNPs have a promising future in antibacterial treatment, and it may be the first option for antibacterial therapy in clinical practice.

## Data Availability

The original contributions presented in the study are included in the article/Supplementary Material; further inquiries can be directed to the corresponding author.

## Glossary

^1^PS: PS in a first excited state
^3^PS: Triplet-state PS
APDT: Antibacterial photodynamic therapy
CDT: Chemodynamic therapy
CR: Cross relaxation
CS: Cooperative sensitization
CSU: Cooperative sensitization upconversion
E. coli: *Escherichia coli*
EMU: Energy migration upconversion
EPS: Extracellular polymeric substance
ESA: Excited-state absorption
ETU: Energy-transfer upconversion
F. nucleatum: *Fusobacterium nucleatum*
HOMO: Highest occupied molecular orbital
LPS: Lipopolysaccharides
MB: Methylene blue
MDR: Multi-drug-resistant bacteria
MIC: Minimum inhibitory concentration
MSSA: Methicillin-sensitive *Staphylococcus aureus*
MSSE: Methicillin-sensitive *Staphycoccus epidermidis*
NIR: Near-infrared
O2−: Superoxide anion
OC: N-octyl chitosan
−OH: Hydroxyl radicals
P. gingivalis: *Porphyromonas gingivalis*
PA: Photoacoustic
PA: Photon avalanche
PAA: Polyacrylic acid
PPT: Photothermal therapy
PS: Photosensitizer
PSeV: Poly(selenoviologen)
PVP: Polyvinyl pyrrolidone
ROS: Reactive oxygen species
S. aureus: *Staphylococcus aureus*
SDT: Sonodynamic therapy
UCL: Upconversion luminescence
UCNPs: Upconversion nanoparticles
UV: Ultraviolet
XRD-AB: Drug-resistant *Acinetobacter baumannii*
